# Child Supervision and Burn Outcome among Admitted Patients at Major Trauma Hospitals in the Gambia

**DOI:** 10.3390/ijerph14080856

**Published:** 2017-07-30

**Authors:** Edrisa Sanyang, Corinne Peek-Asa, Tracy Young, Laurence Fuortes

**Affiliations:** 1 Injury Prevention and Research Center, College of Public Health, University of Iowa, Iowa City, IA 52240, USA; corinne-peek-asa@uiowa.edu (C.P.-A.); tracy-young@uiowa.edu (T.Y.); 2 Department of Public & Environmental Health, School of Medicine & Allied Health Sciences, University of The Gambia, Brikama, PO Box 3530 Serrekunda, The Gambia; 3 Center for International Rural and Environmental Health, College of Public Health, University of Iowa, Iowa City, IA 52240, USA; laurence-fuortes@uiowa.edu

**Keywords:** burns, LMICs, child supervision, cooking, burn outcome

## Abstract

Burn-related injuries are a significant burden in children, particularly in low- and middle-income countries (LMICs), where more than 90% of burn-related pediatric deaths occur. Lack of adult supervision of children is a major risk for pediatric burn injuries. The goal of this paper was to examine the general characteristics of burns and identify burn injury outcomes among adult-supervised children compared to those who were not supervised. The study examined burn injury and clinical characteristics among all burn patients admitted to two trauma hospitals in The Gambia, West Africa. At intake in the emergency room, the treating physician or nurse determined the need for admission based on body surface area burned (BSAB), depth of burn, and other clinical considerations such as co-occurring injuries and co-morbidities. During the study period of 1 April 2014 through 31 October 2016, 105 burn patients were admitted and data were collected by the treating physician for all of them. Information about supervision was only asked for children aged five years or less. More than half (51%) of the burn patients were children under 18 years, and 22% were under 5 years. Among children under five, most (86.4%) were supervised by an adult at the time of burn event. Of the 19 supervised children, 16 (84.2%) had body area surface burned (BSAB) of less than 20%. Two of the three children without adult supervision at the time of burn event had BSAB ≥ 20%. Overall, 59% of the patients had 20% + BSAB. Females (aOR = 1.25; 95% CI = 0.43–3.62), those burned in rural towns and villages (aOR = 2.29; 95% CI = 0.69–7.57), or burned by fire or flames (aOR = 1.47; 95% CI = 0.51–4.23) had increased odds of having a BSAB ≥ 20%, although these differences were not statistically significant. Children 0–5 years or 5–18 years (aOR = 0.04, 95% CI = 0.01–0.17; aOR = 0.07, 95% CI = 0.02–0.23, respectively) were less likely to have BSAB ≥ 20% than adults. Those burned in a rural location (aOR = 9.23, 95% CI = 2.30–37.12) or by fire or flames (aOR = 6.09, 95% CI = 1.55–23.97) were more likely to die. Children 0–5 years or 5–18 years (aOR = 0.2, 95% CI = 0.03–1.18; aOR = 0.38; 95% CI = 0.11–1.570, respectively) were less likely to die. Children constitute a significant proportion of admitted burn patients, and most of them were supervised at the time of the burn event. Supervised children (compared to unsupervised children) had less severe burns. Programs that focus on burn prevention at all levels including child supervision could increase awareness and reduce burns or their severity. Programs need to be designed and evaluated with focus on the child development stage and the leading causes of burns by age group.

## 1. Introduction

Globally, an estimated 180,000 people die each year from burns, and non-fatal burn injuries are a leading cause of morbidity [1]. Burn incidence and related deaths are disproportionately concentrated in low and middle-income countries (LMICs) [2,3]. While high income countries (HICs) are generally experiencing a decrease in burn incidence and burn death [4,5,6], burn cases in LMICs are on the rise, particularly among children [7,8,9]. In the World Health Organization (WHO) African Region for instance, children under five years have almost three times the incidence of burn deaths than children under five years worldwide. Child deaths from burns is currently estimated at seven times higher in LMICs than HICs [2]. Non-fatal burns are a leading cause of prolonged hospitalization, disability, pain, as well as long-lasting stigma and rejection.

Burns occur mainly in the home and workplace. Compared to LMICs, high income countries have made successful progress in lowering home burn injury incidence through the introduction of smoke detectors and overall development of safer buildings, household fuels, and appliances. Workplace burn reductions have been achieved through safety engineering, policy and regulation, and growth in occupational safety culture. This basic infrastructure development in LMICs is lacking [10,11]. Housing standards on fire protection and their enforcement for both home and industries are limited or absent. Major risk factors for burns in LMICs include use of fuel wood for cooking and heating, indoor open fires, and ground-level cookstoves; use of kerosene (paraffin) as a fuel source for non-electric domestic appliances; and inadequate safety measures for liquefied petroleum gas and electricity. Children and women are disproportionately affected [2]. With electricity out most of the time, and less purchasing power for food preservation/refrigeration, the cycle of cooking for most households in The Gambia is still three times a day (for breakfast, lunch, and dinner) and seven days a week, conducted almost entirely through indoor open fires and cookstoves. The repeated exposure to fire puts women and children at increase risk for burns. The higher risk for females is also associated with open fire cooking, or inherently unsafe cookstoves, which can ignite loose clothing [12]. Open flames used for heating and lighting also pose risks, and self-directed or interpersonal violence are also factors, although understudied. Along with adult women, children are particularly vulnerable to burns [2,12]. Burns are the 11th leading cause of death in children aged 1–9 years and are also the 5th most common cause of non-fatal childhood injuries, and increased child hospitalization [2]. While the majority of the risk could be associated with improper adult supervision, some child related burns result from child maltreatment [13,14].

Burn injuries are a multifaceted issue. In addition to risks for burn injury, access to timely, specialized burn care is also critical. The healthcare sector plays an important role in reducing the burden of burn injuries, and currently specialized burn treatment in The Gambia exists in only two major urban hospitals: Edward Francis Small Teaching Hospital and Serrekunda General Hospital. Although the hospitals are large and treat most of the critically ill burnt patients, they lack basic surgical equipment and personnel to adequately care for the overwhelming volume of burn patients [15].

One major contributing factor to Gambia’s inefficiency in addressing burn-related morbidity and mortality has been the inability to collect useful, reliable, and timely data that can be used to implement prevention strategies. Without such data, projections for healthcare provider needs, as well as legislative and policy responses, have been largely absent. In order to build a data infrastructure in The Gambia, the University of The Gambia, in partnership with the University of Iowa Injury Prevention Research Center and the World Health Organization (WHO) piloted a global burn registry in the two major trauma hospitals in The Gambia. This pilot provides Gambia’s first burn injury data using a hospital-based trauma study in the country’s major burn treatment hospitals. This study identifies risk factors and mechanisms of burn injuries among inpatients at two major trauma hospitals in The Gambia. Prior to this study, there was incomplete documentation for burn cases, and mechanisms of burn injuries were largely unknown. One study goal was to explore burn severity among children supervised compared to those unsupervised by adults. The study also examined the general demographic characteristics of burn patients and clinical burn characteristics, and identified factors associated with burns and burn injury outcomes.

## 2. Materials and Methods

Lack of surgical capacity for burn patients has previously been shown to be one of the major barriers to adequate health care in many low-income countries. Burns potentially comprise a large portion of surgical conditions that require operative therapy in low income settings. Only two hospitals in The Gambia—Edward Francis Small Teaching Hospital (EFSTH) and Serrekunda General Hospital (SGH)—have full capability to treat complex trauma cases, and these two hospitals were included in this pilot project. Both are located in Banjul, the major urban center of The Gambia.

The study included all burn patients admitted to EFSTH and SGH from 1 April 2014 to 31 October 2016 (*n* = 105). Edward Francis Small Teaching Hospital (EFSTH) is the largest hospital in The Gambia, and serves as the teaching and referral hospital for the entire country. Serrekunda General Hospital (SGH) is newer and smaller but has similar functions. Together, the two hospitals serve the routine health care needs of the Greater Banjul Area, the only urban area with 59% of the country’s population [16]. These hospitals also receive most of the complex trauma cases throughout the country. We performed analyses of admitted burn patients using the World Health Organization (WHO) Global Burn Registry format.

At intake in the emergency room, the treating physician or nurse determined the need for admission based on percent body surface area burned (BSAB), depth of burn, and other clinical considerations such as co-occuring injuries and co-morbidities. Adults with greater than 15% BSAB and any child under five years of age regardless of burn size were considered for admission. Other considerations for admission included any full thickness burn; burns to face, hands, feet, and perineum; circumferential burns; inhalation injury; and whether an associated trauma or significant pre-burn illness was evident.

At the time of admission, the treating physician completed the Global Burn Registry Data Collection Form developed by the WHO and a global network of burn experts. The 35-item questionnaire described the burned individual and characteristics of the burn. Data were collected on a paper form and retrieved at the hospital site twice a week by the study coordinator. Data was entered into DataCol, a WHO designed electronic platform for data entry. Data entered into the DataCol is only accessible to the WHO administrator and study coordinator by username and password.

We focused on 10 variables in our analysis: age, gender, related cause of burn, place of burn, date and time of burn, primary cause of burn, percent body surface area burned, discharge status from hospital admission, length of hospitalization, and child supervision by adult. Based on the age distribution and objective to examine child supervision by an adult, we collapsed age into three categories: under 5 years (22%), 5–18 (29%), and 19+ years (49%).

Supervision was collected for children under age five. Supervision was categorized as with an adult (including with an adult but not supervised, and with an adult and supervised), and without an adult (including alone, with other children, and unknown). We examined two binary outcome variables: burn severity and hospital survival (died or survived). Burn severity was measured as body surface area burned (% BSAB) and was dichotomized into <20% BSAB and 20% + BSAB. Less than 20% BSAB was categorized as less severe and it corresponds with Abbreviated Injury Scale of 3 or less (AIS ≤ 3); while more severe burns (20% + BSAB) corresponds with AIS of 4 or greater (AIS ≥ 4). Those who died included patients who died of burn-related injuries during hospital admission. Patients categorized as having survived were either discharged, transferred to another facility, or left against medical advice.

Distribution of primary variables were examined by age and % BSAB to identify general characteristics and trends using chi-square and Fisher exact tests. Simple multivariate logistic regression models were used to identify the association of demographic and burn injury characteristics using % BSAB (<20% vs. 20%+) and burn injury outcome (died or alive). All eight variables were examined for inclusion in the logistic models, yet not all could be included due to insignificance in the univariate models and/or small cell sizes. Gender was not significant for either outcome yet was included in both models as an important confounder. Primary cause of burn was also nonsignificant in the univariate model when looking at BSAB outcome yet was clinically relevant to keep in the multivariate model. All analyses were performed using SAS 9.4 (SAS Institute, Inc., Cary, NC, USA).

Ethical approval was obtained from the joint Gambia Government and Medical Research Council Ethics Committee (R13 014v2). Data collection was done in accordance with the Helsinki Declaration. A written consent was obtained from the patients who were mentally alert to respond to questions. The escorts of the unconscious burn patients, who had knowledge about the burn, were asked for consent and interviewed as proxies.

## 3. Results

Among the 105 burn patients treated at Edward Francis Small Teaching Hospital and Serrekunda General Hospital (from 1 April 2014 to 31 October 2016), about a quarter (22%) were children 0–5 years; and more than half (51%) were under 18 years. As shown in Table 1, cooking-related burns were the most common mechanism of burn injuries with 75.5% being children. Unlike other age groups, the proportion of cooking-related burn injuries among children 0–5 years (86.4%) were higher than those 5–18 years (72%) and 19+ years (30%). The proportion of burns by gender for all ages was higher among males than females. An urban location was the most common place for burn injuries (74%). The proportion of burns by time of day for all ages was higher during the daytime than at night. Notwithstanding, the number of daytime burns among children 5–18 years old was only 3.1 times more than those at night, but 8.8 and 4.5 times higher among adults (19+ years) and younger children (0–5 years), respectively. The proportion of children, 0–5 years (86.4%) and 5–18 years (72%), who had cooking-related burns was higher than those with occupational and other-related burns for both age groups. The proportion of adults (19+ years) with occupational-related (55%) burns, however, was higher than cooking-related (30%), and other-related (15%) burns. Though cooking-related (58%) burns were common compared to occupational (27%) and other-related burns (15%), they were less severe (Table 2). Among patients with <10% BSAB, the majority (88%) had cooking-related burns. Seven of the eight patients with 90% + BSAB were involved in an occupational activity (excluding cooking) at the time of the burn event.

Overall, cause of burns for all ages was more often by hot liquid, steam, gas, or hot surface (61%) than by fire or flame (38%). The proportion of children 0–5 years treated for hot liquids, steam, gas, or hot surface burns (73%) was higher compared to those treated for fire or flames (27%), and this high proportion was not found among older children (5–18 years) and adults (19+ years). Among children up to five years of age, 86.4% were supervised or were with adults at the time of the burn. Almost all children with adults had <10% BSAB (100%), 10–19% BSAB (88.9), or 20–29% BSAB (100%). Two of the three children without an adult at the time of burn had 20% + BSAB, which indicates a very severe burn, compared with only 3 of 19 children without an adult. Although the number of children without an adult was too small to estimate an odds ratio, this finding warrants further investigation.

Overall, 59% of the patients had >20% BSAB (Table 3). Females (aOR = 1.25; 95% CI = 0.43–3.62), those burned in rural towns and villages (aOR = 2.29; 95% CI = 0.69–7.57), or burned by fire or flames (aOR = 1.47; 95% CI = 0.51–4.23) were more likely to have a BSAB ≥ 20%. Children 0–5 years or 5–18 years (aOR = 0.04, 95% CI = 0.01–0.17; aOR = 0.07, 95% CI = 0.02–0.23, respectively) were less likely to have >20% BSABs.

About a fifth (21.4%) of the burn patients died in the study hospitals (Table 4). Those burned in a rural location (aOR = 9.23, 95% CI = 2.30–37.12) or by fire or flames (aOR = 6.09, 95% CI = 1.55–23.97) were more likely to die. Children 0–5 years or 5–18 years (aOR = 0.2, 95% CI = 0.03–1.18; aOR = 0.38; 95% CI = 0.11–1.570, respectively) were less likely to die.

## 4. Discussion

To our knowledge, this study is the first report from The Gambia to examine inpatient burns using a systematic data collection method. The study provides epidemiologic characteristics of inpatient burns and burn outcomes in two major trauma hospitals in the country: Edward Francis Small Teaching Hospital and Serrekunda General Hospital. The two hospitals are located in the urban Greater Banjul Area. Since both hospitals were urban, they are likely to see a disproportionate number of urban burns, and this does not represent a population risk.

Our findings suggest that more than half (51%) of the burn patients were under 18 years of age and about a quarter (22%) were under 5 years of age. This is consistent with similar studies in other LMICs including Ghana, Kenya, and Bangladesh [17,18,19]. Irrespective of setting, children are naturally curious and want to explore their environment as soon as they are mobile. This study does not explore what specific activities children were doing leading to the burn event. However, their natural learning process and exploration in home environments with open fires and cookstoves introduces burn risk. This corroborates with our finding where the proportion of children 0–5 years treated for hot liquid, steam, gas, or hot surface burns were 167% higher than those treated for fire or flame burns. This is consistent with similar studies in other low and middle income countries [12,20]. Heating and lighting sources and cooking are major risk factors for burns, especially in LMICs. These high risk energy sources are important to daily living in LMICs, and therefore the need to develop engineering and behavioral interventions that promote safe use of fuel wood for cooking, heating, and light sources or that identify alternative energy sources are critical. In averting cooking-related burns for instance, interventions to develop and promote safe low technology equipment as substitutes for open flames or wood cookstoves that reduce potential child contact to hot objects or limit access to the cooking area could reduce burn incidence and avert burn-related deformities and disabilities [21,22].

Our data suggest that cooking-related activities are a major source of burns. This is consistent with similar studies in Sub-Saharan Africa [1,22,23]. In The Gambia, almost all the domestic work—especially that which is related to food preparation—is done by women and girls. With frequent electricity outage and limited access to refrigeration, cooking with open flames in typical households is done three times a day. Additionally, extensive use of fossil fuel for cooking and the inherent nature of cooking areas poses major risks for burns. Cooking is often done in open places and on the ground with no safety barriers to fire/flames or hot objects on the fire. In congested buildings, separation of the dwelling and cooking areas can be a considerable challenge. This situation is very typical in the Greater Banjul Area, where almost 60% of The Gambia’s population live in 1% of the land [15] with an average household consisting of 8.2 persons [24]. This ushers in overcrowding, resulting in limited space for cooking and dwelling. Cooking taking place in the child's living and playing space especially at ground level, increases the risk of burns.

Our finding suggests that many children may have access to hazardous places in the presence of an adult. Parents may have poor knowledge of burn risks or approaches to prevent burns. In The Gambia, most of the cooking is done in unprotected areas without child safety facilities to limit children having access to fire/flames or even hot cooking utensils.

Numerous interventions have shown that improved cookstoves can reduce burn injuries [20,22,23]. In a case control study in Guatemala, the burn incidence two years previous was 42.1 per 1000 person-years. After the improved cookstove intervention, the control group had 35.2 per 1000 person-years compared to 18.1 per 1000 person-years in the intervention group [20]. However, other studies have shown that even where such child safety facilities exist, such as improved cookstoves, some women still prefer cooking on kitchen floors over using the improved cookstove [25,26,27].

Previous studies on child injury have indicated that child injuries are often the interplay between family, environment, and individual factors [23,24]. Child neglect or supervisory neglect has been associated with child maltreatment and injury [13]. Large families with history of domestic violence or parents who have poor mental health and substance abuse issues are more likely to be associated with poor supervision or lack of awareness of risks for their children. The caveat is that those data are mostly from high income countries and may bear less relevance to LMICs because of safer technologies. Although there are clear differences in burns in this study, details of parents/caregivers supervision are not available to help explain the circumstances in detail. However, it is certain that burn patterns will change based on how parents/caregivers perceive risky environments or risks children pose based on their behaviors. Parental behaviors may be modified as they learn and recognize these changes in the environment, and understand their children’s developmental stages and associated risks.

In general, burn prevention strategies—especially for children—are very low or almost non-existant in LMICs, including The Gambia. Though compared to adults, burns to children might be less severe, yet the disfigurement, life long disability, and potential for child neglect and maltreatment are likely.

This study has several limitations. Data were collected from the two major trauma hospitals in the country, and both are located in the major urban area. Thus, these data reflect all admitted burns in the catchment area of these hospitals. However, patients from rural areas may be treated locally, or in the case of very severe burns may not survive transfer to hospital. This sample is thus not generalizable to the population of the entire country. Since our data show that the number of burn patients was higher for urban than rural areas, yet rural burn patients were had more severe burns, this indicates that this representation is likely present. This new hospital data collection system provided data on all documented burn patients who were admitted to the hospital. However, since the hospitals do not have electronic data systems, an unknown but likely very small number of burn cases may have been missed.

This study was also limited to the size of the pilot data available in the trauma registries at EFSTH and SGH. Some variables were collapsed to have enough cells to predict outcome of interest. Meaning it is possible that some details like burn injury mechanisms and age-specific injury outcome may not be present, therefore introducing inconsistencies. Despite these limitations, the results from this study encourage further investigation of risk for burns, including the potential for child abuse.

## 5. Conclusions

In The Gambia, like other low income countries, children constitute an important proportion of burn patients despite adult supervision at the time of burn events. Most of the burns were cooking related. This could be due to the nature of cooking areas which generally do not provide protection to children, nor are they usually separate from a child’s playing/living area. It could also be due to lack of awareness on the devastating effects of burns and how easily they can occur and affect child health and wellbeing. Considering this result, there is a need to focus on child burn prevention, including advocacy and parent/caregiver education programs, and development and promotion of safer technologies. These programs need to be designed and evaluated with focus on the child development stages and education of parents/caregivers on the potential for lapses in supervision and possible unintentional injuries. When parents/caregivers have information about the burn risks associated with increased mobility and independence of children, they could be able to identify those exposures and avert the burns or reduce burn severity.

## Figures and Tables

**Table 1:** Burn event factors by age ^1^.

Factors	Age								*p*-Value ^4^
	Total		<5		5–18		19+		
	*n*	(%)	*n*	(%)	*n*	(%)	*n*	(%)	
Gender									0.4368
Male	63	(63.0)	16	(72.7)	16	(55.2)	31	(63.3)	
Female	37	(37.0)	6	(27.3)	13	(44.8)	18	(36.7)	
Time of day									0.2402
8:00 a.m.–7:59 p.m.	84	(84.0)	18	(81.8)	22	(75.9)	44	(89.8)	
8:00 p.m.–7:59 a.m.	16	(16.0)	4	(18.2)	7	(24.1)	5	(10.2)	
Place of injury									0.3003
Rural	26	(26.0)	5	(22.7)	5	(17.2)	16	(35.6)	
Urban	74	(74.0)	17	(77.3)	24	(82.8)	33	(64.4)	
Primary cause of burn ^2^									0.3479
Thermal (fire/flame)	38	(38.0)	6	(27.3)	10	(34.5)	22	(44.9)	
Other thermal (hot liquid/steam/gas/surface)	61	(61.0)	16	(72.7)	18	(62.1)	27	(55.1)	
Related cause of burn									<0.0001
Cooking	49	(56.3)	19	(86.4)	18	(72.0)	12	(30.0)	
Occupational activity	25	(28.7)	0	(0.0)	3	(12.0)	22	(55.0)	
Other	13	(14.9)	3	(13.6)	4	(16.0)	6	(15.0)	
Under 5 supervised at time of burn									NA ^3^
With an adult	19	(86.4)	19	(86.4)	0	(0.0)	0	(0.0)	
Without an adult	3	(13.6)	3	(13.6)	0	(0.0)	0	(0.0)	
Length of stay (LOS)									0.2622
0–3 days	36	(36.4)	7	(31.8)	9	(32.1)	20	(40.8)	
4–7 days	29	(29.3)	8	(36.4)	5	(17.9)	16	(32.7)	
8 + days	34	(34.3)	7	(31.8)	14	(50.0)	13	(26.5)	

^1^ Not all variables add up to 105 due to missing patient variables; ^2^ Primary cause = 'Electrical' was omitted due to small sample (*n* = 1); ^3^ No statistics computed due to zero cells; ^4^ *p*-values are from chi-square tests of independence.

**Table 2:** Environmental/adult supervision, and LOS by Body Surface Area Burned ^1^.

Factors	% Body Surface Area Burned (BSAB)														
	Total		<10		10–19		20–29		30–39		40–89		90+		
	*n*	(%)	*n*	(%)	*n*	(%)	*n*	(%)	*n*	(%)	*n*	(%)	*n*	(%)	*p*-Value ^3^
Age															<0.0001
<5	22	(22.0)	8	(47.1)	9	(36.0)	3	(25.0)	1	(14.3)	1	(3.6)	0	(0.0)	
5–18	29	(29.0)	9	(52.9)	10	(40.0)	5	(41.7)	1	(14.3)	1	(3.6)	3	(27.3)	
19+	49	(49.0)	0	(0.0)	6	(24.0)	4	(33.3)	5	(71.4)	26	(92.9)	8	(72.7)	
Gender															0.4761
Male	64	(8.0)	12	(70.6)	15	(57.7)	7	(50.0)	4	(44.4)	17	(60.7)	9	(81.8)	
Female	41	(39.0)	5	(29.4)	11	(42.3)	7	(50.0)	5	(55.6)	11	(39.3)	2	(18.2)	
Time of day															0.4309
8:00 a.m.–7:59 p.m.	16	(15.2)	5	(29.4)	3	(11.5)	1	(7.1)	0	(0.0)	5	(17.9)	2	(18.2)	
8:00 p.m.–7:59 a.m.	89	(84.8)	12	(70.6)	23	(88.5)	13	(92.9)	9	(100.0)	23	(82.1)	9	(81.8)	
Place of injury															0.055
Rural	28	(26.7)	4	(23.5)	3	(11.5)	3	(21.4)	1	(11.1)	11	(39.3)	6	(54.5)	
Urban	77	(73.3)	13	(76.5)	23	(88.5)	11	(78.6)	8	(88.9)	17	(60.7)	5	(45.5)	
Primary cause of burn ^2^															0.1735
Thermal (fire/flame)	38	(36.2)	7	(41.2)	6	(23.1)	3	(21.4)	2	(22.2)	15	(53.6)	5	(45.5)	
Other thermal (hot liquid/steam/gas/surface)	66	(62.9)	10	(58.8)	20	(76.9)	10	(71.4)	7	(77.8)	13	(46.4)	6	(54.5)	
Related cause of burn															<0.0001
Cooking	53	(57.6)	14	(87.5)	21	(91.3)	9	(64.3)	4	(50.0)	5	(21.7)	0	(0.0)	
Occupational activity	25	(27.2)	0	(0.0)	1	(4.3)	1	(7.1)	1	(12.5)	15	(65.2)	7	(87.5)	
Other	14	(15.2)	2	(12.5)	1	(4.3)	4	(28.6)	3	(37.5)	3	(13.0)	1	(12.5)	
Under 5 supervised at time of burn															0.0175
With an adult	19	(86.4)	8	(100.0)	8	(88.9)	3	(100.0)	0	(0.0)	0	(0.0)	0	(0.0)	
Without an adult	3	(13.6)	0	(0.0)	1	(11.1)	0	(0.0)	1	(100.0)	1	(100.0)	0	(0.0)	
Length of stay (LOS)															0.0169
0–3 days	36	(34.6)	4	(23.5)	7	(28.0)	5	(35.7)	2	(22.2)	9	(32.1)	9	(81.8)	
4–7 days	29	(27.9)	6	(35.3)	6	(24.0)	2	(14.3)	1	(11.1)	12	(42.9)	2	(18.2)	
8+ days	39	(37.5)	7	(41.1)	12	(48.0)	7	(50.0)	6	(66.6)	7	(25.0)	0	(0.0)	

^1^ Not all variables add up to 105 due to missing patient variables; ^2^ Primary cause = “Electrical” was omitted due to small sample (*n* = 1); ^3^ *p*-values are from chi-square tests of independence.

**Table 3:** Multivariate logistic regression predicting burn severity ^1^.

Factor	Total		<20% BSAB		20% + BSAB		cOR (95% CI)	aOR (95% CI)
	*n*	(%)	*n*	(%)	*n*	(%)		
Age								
<5	22	(22.0)	17	(40.5)	5	(8.6)	0.04 (0.01–0.15)	0.04 (0.01–0.17)
5–18	29	(29.0)	19	(45.2)	10	(17.2)	0.07 (0.02–0.23)	0.07 (0.02–0.23)
19+	49	(49.0)	6	(14.3)	43	(74.1)	REF	REF
Gender								
Male	64	(61.0)	27	(62.8)	37	(59.7)	REF	REF
Female	41	(39.0)	16	(37.2)	25	(40.3)	1.14 (0.51–2.54)	1.25 (0.43–3.62)
Time of day								
8:00 a.m.–7:59 p.m.	16	(15.2)	8	(18.6)	8	(12.9)	1.54 (0.53–4.49)	NA
8:00 p.m.–7:59 a.m.	89	(84.8)	35	(81.4)	54	(87.1)	REF	REF
Place of injury								
Rural	27	(26.2)	7	(16.3)	20	(33.3)	2.63 (1.00–6.92)	2.29 (0.69–7.57)
Urban	76	(73.8)	36	(83.7)	40	(66.7)	REF	REF
Primary cause of burn ^2^								
Thermal (fire/flame)	38	(36.5)	13	(30.2)	25	(41.0)	1.60 (0.70–3.66)	1.47 (0.51–4.23)
Other thermal (hot liquid/steam/gas/surface)	66	(63.5)	30	(69.8)	36	(59.0)	REF	REF
Related cause of burn								
Cooking	53	(57.6)	35	(89.7)	18	(34.0)	NA	NA
Occupational activity	25	(27.2)	1	(2.6)	24	(45.3)	REF	REF
Other	14	(15.2)	3	(7.7)	11	(20.7)	NA	NA
Under 5 supervised at time of burn								
With an adult	19	(86.4)	16	(94.1)	3	(60.0)	REF	REF
Without an adult	3	(13.6)	1	(5.9)	2	(40.0)	NA	NA
Length of stay (LOS)								
0–3 days	36	(34.6)	11	(26.2)	25	(40.3)	REF	REF
4–7 days	29	(27.9)	12	(28.6)	17	(27.4)	0.62 (0.22–1.74)	NA
8+ days	39	(37.5)	19	(45.2)	20	(32.3)	0.46 (0.18–1.19)	NA

^1^ Not all variables add up to 105 due to missing patient variables; ^2^ Primary cause = “Electrical” was omitted due to small sample (*n* = 1).

**Table 4:** Multivariate logistic regression predicting death ^1^.

Factor	Total		Alive		Died		cOR (95% CI)	aOR (95% CI)
	*n*	(%)	*n*	(%)	*n*	(%)		
Age								
<5	21	(21.4)	19	(25.0)	2	(9.1)	0.22 (0.04–1.05)	0.20 (0.03–1.18)
5–18	28	(28.6)	24	(31.6)	4	(18.2)	0.34 (0.10–1.16)	0.38 (0.11–1.57)
19+	49	(50.0)	33	(43.5)	16	(72.7)	REF	REF
Gender								
Male	62	(60.2)	45	(55.6)	17	(77.3)	REF	REF
Female	41	(39.8)	36	(44.4)	5	(22.7)	0.37 (0.12–1.09)	0.38 (0.11–1.32)
Time of day								
8:00 a.m.–7:59 p.m.	87	(84.5)	69	(85.2)	18	(81.8)	NA	NA
8:00 p.m.–7:59 a.m.	16	(15.5)	12	(14.8)	4	(18.2)	REF	REF
Place of injury								
Rural	28	(27.2)	17	(21.0)	11	(50.0)	3.77 (1.40–10.15)	9.23 (2.30–37.12)
Urban	75	(72.8)	64	(79.0)	11	(50.0)	REF	REF
Primary cause of burn ^2^								
Thermal (fire/flame)	38	(37.3)	25	(31.3)	13	(59.1)	3.18 (1.20–8.40)	6.09 (1.55–23.97)
Other thermal (hot liquid/steam/gas/surface)	64	(62.7)	55	(68.8)	9	(40.9)	REF	REF
Related cause of burn								
Cooking	52	(57.8)	51	(68.9)	1	(6.2)	NA	NA
Occupational activity	24	(26.7)	11	(14.9)	13	(81.2)	REF	REF
Other	14	(15.6)	12	(16.2)	2	(12.5)	NA	NA
Under 5 supervised at time of burn								
With an adult	19	(90.5)	18	(94.7)	1	(50.0)	REF	REF
Without an adult	2	(9.5)	1	(5.3)	1	(50.0)	NA	NA
Body surface area burned (%)								
<20%	42	(40.8)	41	(50.6)	1	(4.5)	REF	REF
20%+	61	(59.2)	40	(49.4)	21	(95.5)	NA	NA
Length of stay (LOS)								
0–3 days	36	(35.0)	20	(24.7)	16	(72.7)	REF	REF
4–7 days	28	(27.2)	24	(29.6)	4	(18.2)	NA	NA
8+ days	39	(37.8)	37	(45.7)	2	(9.1)	NA	NA

^1^ Not all variables add up to 105 due to missing patients; ^2^ Primary cause = “Electrical” was omitted due to small sample (*n* = 1).
